# Anti-malarial drug safety information obtained through routine monitoring in a rural district of South-Western Senegal

**DOI:** 10.1186/1475-2875-11-402

**Published:** 2012-12-05

**Authors:** Philippe Brasseur, Michel T Vaillant, Piero L Olliaro

**Affiliations:** 1 Institut de Recherche pour le Développement (IRD), UMR 198, 1386, BP, Dakar, Sénégal; 2 Methodology and statistics Unit (CCMS) Centre de Recherche Public (CRP)-Santé, Strassen, Luxembourg; 3 UNICEF/UNDP/WB/WHO Special Programme for Research & Training in Tropical Diseases (TDR), 20 avenue Appia, Geneva 27, CH, 1211, Switzerland; 4 Centre for Tropical Medicine and Vaccinology, Nuffield Department of Medicine, University of Oxford, Churchill Hospital, Oxford, UK

## Abstract

**Background:**

Knowing the safety profile of anti-malarial treatments in routine use is essential; millions of patients receive now artemisinin combination therapy (ACT) annually, but the return on information through current systems is as yet inadequate. Cohort event monitoring (CEM) is a WHO (World Health Organization)-recommended practice; testing its performance and feasibility in routine practice in malaria-endemic is important.

**Methods:**

A nine-year CEM-based study of the safety of artesunate-amodiaquine (ASAQ) at five peripheral health facilities in a rural district of South-western Senegal. Staff (nurses, health workers) were trained to collect actively and systematically information on the patient, treatment and events on a purposely designed questionnaire. The occurrence and severity of events was collected before, during and after treatment up to 28 days in order to generate information on all adverse events (AEs) as well as treatment-emerging signs/symptoms (TESS). Laboratory tests (haematology, liver and renal) was planned for at least 10% of cases.

**Results:**

During 2001–2009, 3,708 parasitologically-confirmed malaria cases (mean age = 16.0 ± 12.7 years) were enrolled (26% and 52% of all and parasitologically-confirmed ASAQ treatments, respectively). Treatment was supervised in 96% of cases. Products changed over time: 49% were a loose combination of individually-packaged products (available 2001–03), 42% co-blistered products (2004–09) and 9% a fixed-dose co-formulation (2006–09); dosing was age-based for 42%, weight-based for 58%. AS and AQ were correctly dosed in 97% and 82% of cases with the loose and 93% and 86% with the fixed combination, but only 50% and 42% with the co-blistered product.

Thirty-three per cent (33%) of patients had at least one sign/symptom pre-treatment, 12% had at least one AE and 9% a TESS (total events 3,914, 1,144 and 693, respectively). AEs overestimated TESS by 1.2-2 fold (average 1.7). Changes in laboratory value were insignificant. Over-dosing more than doubled the risk of TESS, though statistical significance was reached only during 2003–2007. The incidence of serious events (including death) was five per thousand.

**Conclusions:**

The study was successful in quantifying and characterizing known reactions and has benchmarking value. Health staff performance varied. Investments in training, motivating and providing a quality control system would be needed. The study proved that a CEM-based system is feasible in this setting but more research is needed to assess whether it is sustainable and what conditions would make it cost-effective, including the amount and quality of data generated, and the use thereof for decision-making.

## Background

Artemisinin-based combination therapy (ACT) is the first-line treatment of uncomplicated malaria today, adopted in 84 of the 87 *Plasmodium falciparum*-endemic countries of the world [1]. While the exact total volume of ACT use is not known, the number of ACT treatment courses procured by the public sector alone in 2010 was 181 million to total more than 600 million treatments since 2005. The main safety liabilities of the drugs involved in ACT are generally well-characterized through information generated by pre-clinical and clinical studies. Now, with widespread use, comes the need for monitoring and evaluation, including drug safety and tolerability. Nevertheless, safety monitoring is challenging in many respects.

The World Health Organization (WHO) encourages countries to set up appropriate pharmaco-vigilance (PV) systems to gather safety information [2]. However, there are practical difficulties in setting-up PV systems in developing countries [3,4]; furthermore, between the clinical trial set-up (selected, closely monitored patients) and passive adverse event reporting in PV (more apt to generate signals about rare events), there is a knowledge gap as to a number of safety and tolerability parameters in real-life settings.

This study was a pilot testing of a clinical and laboratory safety monitoring system, based on cohort event monitoring (CEM), a method that is recommended by the WHO and has been applied to anti-malarials [5,6]. The general objective here was to identify a practice which could be both informative and simple enough for routine use in peripheral health centres – here applied to the safety of the ACT artesunate plus amodiaquine (ASAQ) when administered in real-life conditions.

The specific objectives of this study were to: (i) characterize known reactions (and possibly generate signal on yet unrecognized reactions, depending on the sample size collected); (ii) estimate risks and identify risk factors; (iii) assess adequacy of dosing with different ASAQ formulations and its effects on tolerability; (iv) generate information on the feasibility of such system and possibly point to corrective actions to make it perform better, as needed. This study was not geared to obtain the full spectrum of information that may be generated in CEM studies, such as comparisons with other medications, and a fuller assessment of risks and risk management.

In Senegal, the anti-malarial treatment policy [7] changed from monotherapy (chloroquine or quinine) on clinical diagnosis to amodiaquine (AQ) plus sulphadoxine/pyrimethamine (SP) in 2005 (as an interim measure) and then artesunate plus amodiaquine (ASAQ) for confirmed malaria in 2006. Rapid diagnostic tests (RDT) were made available from 2007 [7].

Worldwide, ASAQ is nowadays the second ACT in terms of volumes procured (41 million in 2010, or 23% of total ACT). In the study area (the district of Oussouye, Casamance), ASAQ was introduced earlier (2000) on a pilot scale and gradually extended. This study followed the various phases of the staggered deployment.

## Methods

### Patients

This study was conducted at the outpatient clinics of four dispensaries (Mlomp, Oussouye, Kabrousse and Djembereng) in the District of Oussouye, Southern Casamance, Senegal. During 2001–2009, patients presenting at these health facilities under routine conditions with fever received an anti-malarial treatment if malaria was suspected on clinical grounds with or without parasitological confirmation. For the purpose of this study, only patients with falciparum malaria confirmed by Giemsa-stained thin and thick blood smear or rapid diagnostic test (RDT, Paracheck, Orchid, India, since 2007) and treated with ASAQ were enrolled. Children under 5kg and pregnant or breast-feeding females, patients not living in the village and not available for the scheduled visits, as well as other anti-malarial treatments, and treatments without parasitological confirmation were excluded.

### Study procedures

Screening and treatment was done by the nurse, parasitological diagnosis by the laboratory technician. Treatment was supervised by the nurse at the health centre (on Days 0, 1 and 2), then the patient was seen again on Day 3 (first day after treatment when a thick blood smear was made for parasitogical assessment) and Day 28 for clinical assessment, or any time in between if needed. Non-attendees for the scheduled clinic visit were actively sought by community health workers. At each visit, the clinical status was assessed and the patient was questioned on the occurrence of signs and symptoms using a pre-established questionnaire; data were recorded by the nurse.

All staff members (nurses and community health workers) were trained at the beginning of the study at the Oussouye district hospital by one of the investigators (PB) as to how to assess, collect and record adverse events on the questionnaire. The questionnaire had been developed by iteration through discussion with the staff and experts. This CEM study was purposely nested within daily routine at the sites. Staff were asked to enrol as many subjects as possible within their capacity and to aim for at least 10% of them to have also haematology, liver and renal functions investigated.

The study was carried out in compliance with the Helsinki Declaration and was approved from the national ethic committee. A written informed consent was obtained from the patients.

### Safety monitoring

Data were recorded in an ad-hoc developed case record form, comprising:

(i) A unique identifier for each patient,

(ii) Demography (age, weight, sex),

(iii) Treatment (product) and dose (Days 0, 1 and 2),

(iv) Parasitological diagnosis (rapid diagnostic test (RDT) and thick smear) and body temperature (Days 0, 1, 2 and 3),

(v) Information (Days 0, 1, 2, 3, 14, and 28) on (a) the presence of a pre-defined set of signs and symptoms to be collected (nausea, vomiting, abdominal pain, diarrhoea, anorexia, asthenia, headache, vertigo, pruritus, cutaneous rash), as well as options for unsolicited signs/symptoms, and (b) event intensity (grading specified from absent = 0, mild =1, moderate = 2, severe = 3, very severe = 4),

(vi) Laboratory data (Days 0, 7, 14 and 28): white cells total counts (WCC), haematocrit (Ht), alanine transaminase (ALT), aspartate transaminase (AST), bilirubin, creatinine (target: at least 10% of all patients treated).

Data were double keyed in Excel® using an end-user formatted sheet with online edit-checks.

### Treatment

Initially in 2001, the use of ASAQ treatment was restricted to the rainy season period, and subsequently extended from 2002 all year round. The target doses are 4 mg / kg / day for AS and 10 mg/ kg / day for AQ. The therapeutic window is 2–10 mg/kg/d (total 6–30 mg/kg) for AS and 7.5-15 mg/kg/d (total 22.5-45 mg/kg not to exceed 600 mg/d or 1800 mg total dose) for AQ [9].

Patients were treated once daily for three days with the following drugs:

● Loose combination of individually-formulated AS and AQ (used during 2000–2003) on a weight-based regimen: Arsumax® 50 mg AS tablets (Sanofi-Aventis, France) 4 mg / kg / day, and Camoquin® 200 mg AQ base tablets (Parke-Davis, Senegal), 10 mg/ kg/day. Tablet fractions were used as appropriate.

● Co-blistered products (Arsucam® used during 2003–2009) dosed either on age or weight containing: Arsumax® (as above) and Flavoquine® 153 mg AQ base tablets (Sanofi-Aventis) or Falcimon® (Cipla, India). Treatment by age was given according to the manufacturer’s instructions: (i) for children < 1 year (weight < 10 kg) = ½ tablet of each drug; (ii) 1 to < 6 years (10–20 kg) = one tablet of each drug; (iii) 6 to < 13 years (21–40 kg) = two tablets of each drug and (iv) 13 years (> 40 kg) = four tablets of each drug.

● Fixed-dose co-formulation (Coarsucam® used during 2006–2009) dosed based on age. The product is available in three different strengths: (i) AS 25 mg and AQ base 67,5 mg, 1 tablet for children 2–11 months (≥ 4.5kg and < 9kg); (ii) AS 50 mg and AQ base 135 mg, 1 tablet for children 1–5 years (≥ 9kg and < 18kg); and (iii) AS 100 mg and AQ base 270 mg, 1 tablet for children 6–13 years (≥ 18kg and < 35kg), and 2 tablets for adults > 14 years (≥ 36kg).

### Statistical methods

Dose of treatment, age and weight were summarized as mean +/− standard deviation. Categorical variables and safety indicators were presented as frequency and percentage. Age was treated both as continuous and categorical variable (age strata: < 6, 6–15, > 15 years).

Adequate dosing assessment was done as per Brasseur *et al*. [8] by determining the proportions of patients within (correctly dosed) or outside (under- or over-dosed) the therapeutic window [9] of the actual dose in mg/day versus the target dose range. The difference between the dose received (in mg/d) outside the therapeutic range and the mean doses of the therapeutic range was also estimated. A logistic model explored possible risk factors for inadequate dosing (outside therapeutic ranges), allowing for age and weight categories, product and year of study. A descending stepwise modelling based on the likelihood ratio test between subsequent models was carried out and two-way interactions were tested.

Safety was assessed by describing (frequency, intensity) (1) events occurring at baseline (pre-treatment); (2) events occurring at any time post-treatment, which were defined as: (2a) adverse events (AE) = any untoward event occurring on or post-treatment independent of causality and (2b) treatment emergent sign/symptom (TESS = events that were not present, or whose intensity was lower, before treatment or worsened with treatment); and (3) laboratory parameters.

Intensities of symptoms were graded as 0–4 (none, mild, moderate, severe and very severe). The common toxicity criteria for adverse events (CTCAE Version 3.0 08/09/2006) were used to evaluate and grade the severity of clinical events and laboratory measurements. The risk of suffering from at least one TESS during the study was estimated with a mixed effects logistic model taking the administration of the different combinations through years as random effect (residual-type random component to specify R-side variance and covariance structures). The model was adjusted on the same variables, selected through a descending modelling procedure with interactions tested thereafter. A *p* value of < 0.05 was considered statistically significant. All tests were two-tailed. Statistical analyses were conducted with the statistical package SAS version 9.2 (SAS Institute, Cary, NC, USA).

## Results

### Baseline characteristics

During 2001–2009, a total of 3,708 patients were enrolled in the study (26% of the total parasitologically-confirmed cases of falciparum malaria treated with ASAQ at these health facilities); female to male ratio = 45:55, mean age = 16.0 ± 12.7 years, mean body weight = 36.5 ± 18.9 kg (Table 1).

**Table 1:** Patients baseline characteristics

	**2001**	**2002**	**2003**	**2004**	**2005**	**2006**	**2007**	**2008**	**2209**	**total**
N treatments	302	653	925	480	462	314	212	222	138	3708
% total	8.1	17.6	25.0	12.9	12.5	8.5	5.7	6.0	3.7	100.00
Female: male (n = 3696)	44:56	46:54	48:52	54:46	43:57	41:59	36:64	37:63	39:61	45:55
Age, yrs mean (SD) (n = 2721)	12.9(10.5)	15.3(12.4)	15.9(13)	15.8(12.2)	13.6(11.6)	17.5(12)	17.8(13.4)	20.6(14.1)	22(14.6)	16(12.7)
Weight, kg mean (SD) (n = 3626)	31(16.5)	36.7(19.6)	37.9(20)	36.5(18.3)	32.2(16.8)	40.4(17.7)	36.2(18)	39.4(17)	47.3(19.3)	36.5(19)

Table 2 summarizes the number of treatments by pharmaceutical form (loose, co-blister or fixed co-formulation products) and dosage (age- or weight-based). The loose combination was given to 1,820 (49%) patients during 2001–03 (99% dosed based on body weight), the co-blistered product to 1,568 (42%) patients during 2003–09 (23% dose on weight, 78% by age), and the co-formulation to 320 (9%) patients from 2006 (all age-based); overall, the dose was calculated on body weight for 2,166 (58%) and on age for 1,542 (42%) patients. Treatment intake was supervised for 3,552 patients (96%) and unsupervised to 156 (co-blistered product in 2005).

**Table 2:** Products and regimens

	**Age-based**	**Weight-based**	**Total**	**%**
Loose	12	1808	1820	49%
Co-blistered	1210	358	1568	42%
Fixed-dose	320	0	320	9%
Total	1542	2166	3708	
%	42%	58%		

The age of the malaria cases recruited in the CEM programme increased over time from a median of 10 (range 6–15, mean 12.9 ± 10.4) to 18 years (range 11–30, mean 21.9 ± 14.6).

Table 3 provides the breakdown of treatments by age strata for the 2,659 patients with age and dosage data. The 6–15 years group had ~50% of patients both overall and for the co-blister and loose combination, but only 36% for the fixed-dose product, reflecting the changes in patients’ age over time. The largest single group was the 6–15 years old on the loose combination (nearly one-third of all cases), also reflecting the decreasing number of cases with time.

**Table 3:** Proportion of study participants in the different age strata

	**Loose**			**Co-blister**			**Fixed-dose**			**Total**	
**Age**	**N**	**% form**	**% tot**	**N**	**% form**	**% tot**	**N**	**% form**	**% tot**	**N**	**% tot**
< 6 years	300	14.0 %	11.3 %	130	18.5 %	4.9 %	18	9.3 %	0.7 %	448	16.8 %
6-15 years	844	47.9 %	31.7 %	369	52.6 %	13.9 %	70	36.1 %	2.6 %	1283	48.3%
> 15 years	619	35.1 %	23.3 %	2.3	28.9 %	7.6 %	106	54.6 %	4.0 %	928	34.9 %
Total	1763		66.3 %	702		26.4 %	194		7.3 %	2659	

### Performance and compliance

**Figure 1:**
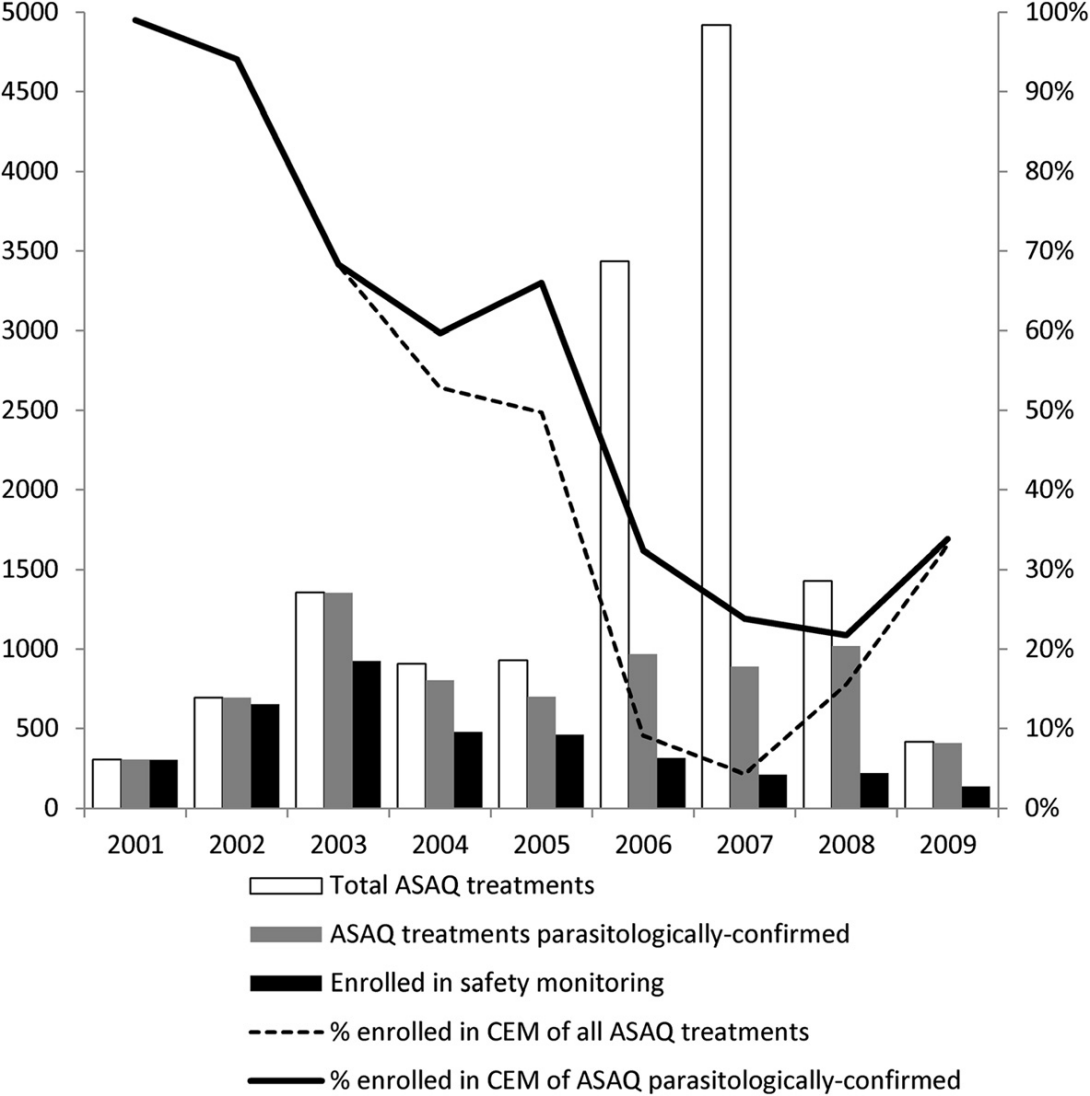
Number and proportion of ASAQ treatments administered and recruited into the study between 2001–2009.

The total number of ASAQ treatments given during 2001–2009 was 14,394 (given on either clinical suspicion or parasitological confirmation), of which 50% (7,144) on parasitological confirmation. Thus, the 3,708 cases enrolled represent 26% of all and 52% of the parasitologically-confirmed ASAQ treatments. Recruitment into the CEM study varied over time. It was very high in 2001 (99%) but decreased steadily through 2007, and only started increasing slightly thereafter. Of note, by 2003, 50% of the total number of cases had been in the CEM study, as compared to 2005 for ASAQ treatments on parasitological confirmation and 2006 for all treatments (Figure 1).

**Figure 2:**
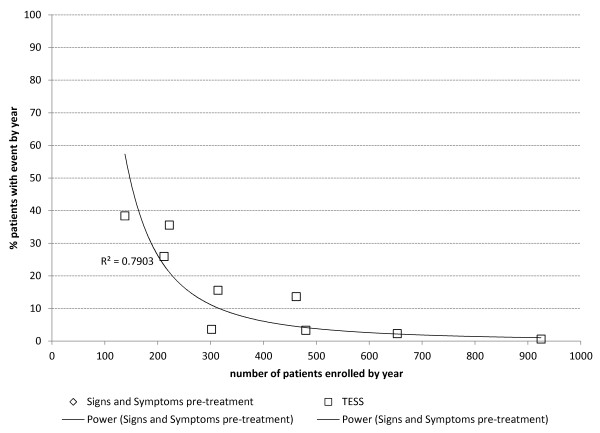
Correlation between the proportions of patients reported to have signs/symptoms on admission or TESS and the number of patients recruited into the study within a year of the study.

There was a strong inverse non-linear correlation between the number of patients recruited in the study in the year and the proportion of patients with recorded signs/symptoms on admission (r^2^ = 0.81) or TESS (r^2^ = 0.79) (Figure 2).

**Figure 3:**
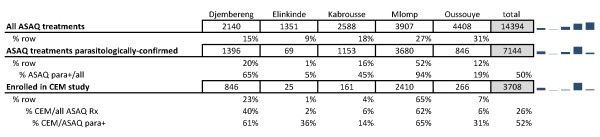
Number and proportion of ASAQ total and parasitologically-confirmed treatments, and enrolments in the CEM study by dispensary.

Of the five sites, Mlomp contributed 62% of the total patients in the CEM evaluation, vs. 52% of the ASAQ treatments on parasitological confirmation and 27% of all ASAQ treatments. Djembereng enrolled 23%, Oussouye 7%, Kabrousse 4% and Elinkinde 1% in the CEM study. This means that Mlomp enrolled in the CEM study 65% of their parasitologically-confirmed ASAQ treatments, Djembereng 61%, Elinkinde 36%, Oussouye 31% and Kabrousse 14%. The respective enrolments are displayed in Figure 3.

### Regimens and adequacy of dosing

Dosage is summarized in Table 4. The overall mean (± standard deviation) dose received over three days was 139 ± 72 mg for AS and 374 ± 184 mg for AQ. Compared to the respective therapeutic windows, AS was correctly dosed in 77% of patients, under-dosed in 5% and over-dosed in 18%, while AQ was correctly dosed in 69% of patients, under-dosed in 9% and over-dosed in 23%. Adequacy of dosage was not the same for all forms. The co-blistered (age-based) products over-dosed both AS (42%) and AQ (48%). The fixed-dose co-formulation (age-based) was as good as the loose co-administration (weight-based) in correctly dosing both AS (97% vs. 93%) and AQ (82% vs. 86%).

**Table 4:** Dosage by product form

	**Loose**		**Co-blister**		**Fixed-dose**		**All**	
	**AS**	**AQ**	**AS**	**AQ**	**AS**	**AQ**	**AS**	**AQ**
Mean total dose mg (SD)	161 (54)	437 (147)	127 (69)	371 (202)	143 (75)	366 (175)	139 (72)	374 (184)
N (%) under-dosed	5 (3%)	20 (10%)	105 (8%)	131 (10%)	71 (4%)	132 (7%)	181 (55)	283 (9%)
N (%) correctly dosed	193 (97%)	163 (82%)	660 (50%)	550 (42%)	1700 (93%)	1570 (86%)	2553 (77%)	2283 (69%)
N (%) over-dosed	0 (0%)	15 (8%)	546 (42%)	630 (48%)	48 (3%)	177 (6%)	594 (18%)	762 (23%)

The OR estimates for being inadequately dosed were significantly higher for the co-blister than the loose (OR 9 95%CI 7.5–10.7) or the fix-dose combination (6.7 (4.5 – 9.8)) while no difference was apparent between the loose and the fixed combinations (0.7 (0.5–1.1)). These differences remained after allowing for age in the analyses. The risk of inadequate dosing was the same across all ages both overall and for each formulation. Compared to the loose product (weight-based), the mean AS dose was ~11% lower with the co-blister and ~13% higher with the fixed product, and the mean AQ dose was about the same with the co-blister but 20% higher with the fixed co-formulation.

**Figure 4:**
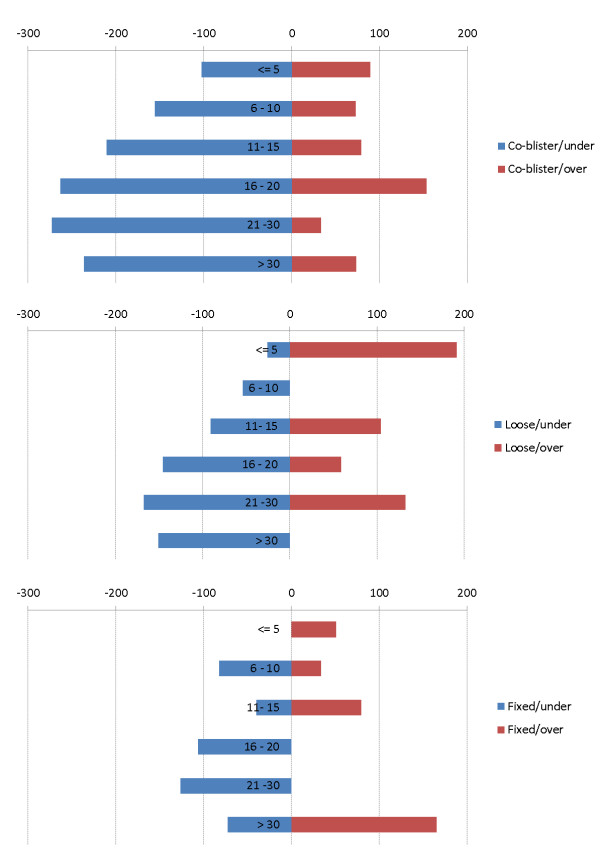
Mean delta (mg AQ) between dose received and lower and upper bound of the therapeutic window (7.5 - 15 mg/kg/d) for patients under and over-dosed, respectively, with the co-blistered, loose and fixed-dose formulation.

Figure 4 shows the size of the mean deviation above or below the respective therapeutic windows for AQ for the three pharmaceutical forms.

### Safety evaluation: adverse events (AEs) and treatment-emergent signs and symptoms (TESS)

Safety records are available for 3,708 patients enrolled during 2001–2009. The number of patients experiencing events and the number of events are summarized in Table 5. At presentation (Day 0, pre-treatment), all patients reported fever or had a measured fever in the clinic. Before treatment start, 1,213 (33%) were reported to experience a total of 3,816 events (signs or symptoms), of which 73% were mild-moderate and 27% severe-very severe.

**Table 5:** Events occurring pre-treatment and on or post-treatment (AE = adverse events; TESS = treatment-emergent signs & symptoms)

	**Pre-treatment**	**On or Post-treatment**	
		**AE**	**TESS**
N patient with at least 1 event	1213	441	347
*(%)*	*33%*	*12%*	*9%*
N patient with at least 1 event by age			
< 6 yr	141 (4%)	35 (8%)	28 (8%)
6-16 yr	529 (44%)	167 (38%)	126 (36%)
> 16 yr	521 (43%)	228 (52%)	175 (50%)
p-value	< .0001	< .0001	< .0001
N of events	3897	1144	688
N mild/ moderate	2828	806	516
*(%)*	*73%*	*70*%	*75*%
N severe/ very severe	1071	338	172
*(%)*	*27%*	*30%*	*25%*

82 events missing in the age-stratified analysis due to missing age.

The risks of both pre- and on/post-treatment events differed depending on age; children under the age of 6 were significantly less likely to have events recorded.

The most common of these malaria-associated signs/symptoms on presentation were headache [n = 990 (25%)], weakness [n = 653 (17%)], vomiting [n = 569 (15%)] vertigo [n = 557 (14%)], nausea [n = 432 (11%)], anorexia [n = 394 (10%)] (Table 6 for all subject, n = 3,708). The number and proportion of events occurring in the three age strata are provided in Table 7 (patients with data on age and events, n = 2,659). Reporting on pre-treatment events varied widely from year to year (from less than 2% in 2003 to > 90% in 2001, 2007, 2008 and 2009).

**Table 6:** Overall number and type of event (n = 3708)

**Events**	**Pre-treatment**	**On or Post-treatment**		**AE:TESS**
		**AE**	**TESS**	
**Diarrhoea**	141	68	57	1.2
**Abdominal pain**	177	71	60	1.2
**Anorexia**	390	70	49	1.4
**Asthenia**	650	116	81	1.4
**Nausea**	431	101	63	1.6
**Headache**	986	233	135	1.7
**Vomiting**	568	239	123	1.9
**Vertigo**	554	246	120	2.1
**Total**	3897	1144	688	1.7

**Table 7:** Number and type of event by age strata

				**On or Post-treatment**								
**Event**	**Pre-treatment**			**AE**			**TESS**			**AE:TESS**		
	**< 6 yr**	**6-15 yr**	**> 15 yr**	**< 6 yr**	**6-15 yr**	**> 15 yr**	**< 6 yr**	**6-15 yr**	**> 15 yr**	**< 6 yr**	**6-15 yr**	**> 15 yr**
**Diarrhoea**	17	50	74	6	22	40	5	18	34	1.2	1.2	1.2
**Abdominal pain**	9	71	94	2	25	42	2	21	35	1.0	1.2	1.2
**Anorexia**	45	152	187	5	18	43	2	14	30	2.5	1.3	1.4
**Asthenia**	87	297	260	8	41	63	4	29	44	2.0	1.4	1.4
**Nausea**	40	226	157	7	45	43	2	29	27	3.5	1.6	1.6
**Headache**	107	436	424	15	82	122	10	47	68	1.5	1.7	1.8
**Vomiting**	69	292	199	27	98	101	11	40	57	2.5	2.5	1.8
**Vertigo**	46	224	274	9	79	145	3	43	65	3.0	1.8	2.2
**Total**	421	1749	1669	79	410	599	39	241	360	2.0	1.7	1.7

Due to missing data on age, numbers of events are not the same as in (a) Table 6 (n = 2659).

After treatment, 441 patients (12%) experienced 1144 AEs (70% mild-moderate; 30% severe-very severe). For 347 of these patients, the event was either not present pre-treatment, or worsened post-treatment (TESS); the total number of TESS was 688 (75% mild-moderate; 25% severe-very severe).

Recording and grading events pre-treatment allowed correcting safety evaluation; AEs overestimated the risk of toxicity as compared to TESS by ~70% on average (range 20% for diarrhoea to doubling for vomiting and vertigo) (Tables 6 and 7).

**Figure 5:**
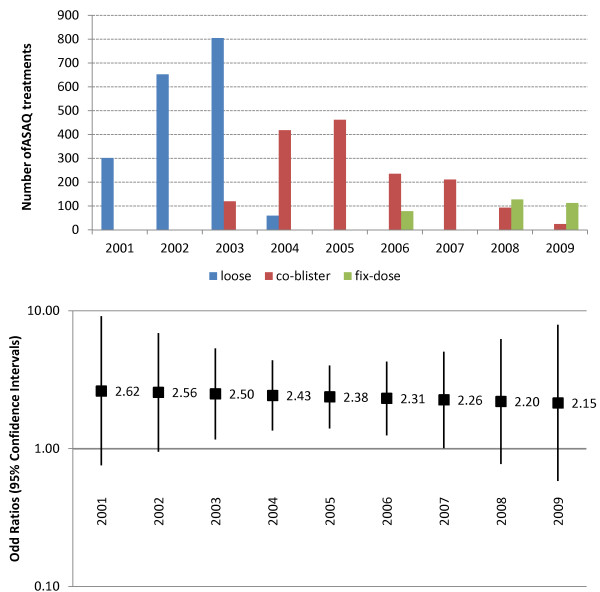
Use of different ASAQ forms (upper panel) and risk of TESS with over-dosing (bottom panel) expressed as OR (95%CIs).

It was not possible to estimate properly the risks of TESS in different groups (age, year of study, or treatment) because of multiple interactions: the age of patients increased over time, while the different products were being deployed at different times; health worker’s compliance with the protocol also changed over time (see below). It was however possible to estimate the risk of TESS with respect to the adequacy of dosing for whole years (whatever the treatment). Figure 5 presents the number of treatments by ASAQ form (loose, co-blistered and fixed co-formulation) and the risk of TESS with over-dosing relative to correct dosing. The relative risk of TESS could be approximated by using odds ratio (OR with 95%CI) as the frequency of the event was limited (193/2,570 = 7.5% patients presenting at least one TESS). While the OR for TESS was always more than twice as big in case of over-dosing, the difference was statistically significant (lower bound of the CI > 1) only during 2003–2007, which coincided with years during which the co-blistered product was the predominant or the only product in use. In contrast, no excess risk was detected in 2001–2002 (only loose combination used) and 2008–2009 (predominantly fixed product).

### Serious adverse events

A total of 19 serious adverse events (SAEs) were recorded: 15 cases requiring hospitalization (ASAQ treatment interrupted and replaced by quinine), two cases of tongue protrusion (ASAQ treatment not interrupted, symptomatic treatment administered), and two deaths.

Fifteen patients, eight treated with the co-blistered product (one weight-based and seven age-based) and seven treated with the co-formulation, were withdrawn from the study because of an adverse event for an overall, crude withdrawal rate of 0.9%: 3.8% [(co-blister or co-formulation) vs. 0% (loose), p < 0.0001. Among these 15 patients, 12 were withdrawn for vomiting (seven treated with the co-blistered and five with co-formulated product) and three for profound asthenia (one treated with co-blister and two with co-formulation). All events were considered probably related to ASAQ except two cases of vomiting (possibly related). In 5/15 cases, the daily dose of AQ exceeded the target dose by 20% or more. All 15 patients were admitted to hospital for intravenous quinine and symptomatic treatment and all recovered without sequelae.

Two other patients were also admitted to hospital for involuntary tongue protrusion. Symptom appeared in a 15-year old patient at D1, 10 hours after the second intake of AS/AQ (co-blister) and disappeared 30 min after administration of diazepam (0.2 mg/kg) IM. Treatment by ASAQ was not discontinued and the patient received a total dose of 612 mg of AQ over three days. Amodiaquine and desethyl-amodiaquine plasma concentrations 90 min after the third drug intake were 8ng/ml and 244ng/ml respectively. The patient completely recovered and was followed up until D28.The parents of this child reported having given a traditional remedy in addition to the medical one but no information could be obtained on this type of treatment. The second case was a five-year old patient who was admitted to hospital for involuntary tongue protrusion two hours after the second intake of AS/AQ (co-blister) and this symptom disappeared 15 min after administration of dexamethazone (0.2 mg/kg) IM. The patient completely recovered and was followed-up for 28 days.

Two patients died. The first one was a three-year old girl attending the health post for a 37°9 fever. Parasitaemia was 164,120/μL asexual *P*. *falciparum* parasites. She was treated with the co-blistered product (50 mg AS and 153 mg AQ base for three days); fever and parasites disappeared on D1. She died at D7 after suffering a traffic injury. The second was an 11-year old girl with fever and a *P*. *falciparum* positive smear. She was treated with a co-blistered product (100 mg AS and 306 mg AQ base) for three days. Fever and parasites disappeared on Day1 and 3, respectively. In the evening of Day 4 the child was found dead in her great mother home with severe dehydration.

### Laboratory investigations

Pre-treatment results were available in 10% of patients for haematocrit, 9% for total WBC, 7% for ASAT and ALAT, 6% for creatinine and 4% for bilirubin. There were no clinically significant changes in mean values between Day 0 and post-treatment (D7-28) except a decrease in ALAT and ASAT (Table 8). No CTC grade 4 values were present at any time. Grades decreased between Day 0 and Days 7 and 28 post-treatment (Table 9).

**Table 8:** Laboratory parameters assessed pre-treatment (Day 0, baseline (BL) and post-treatment (Days 7, 14, 28)

	**BL**			**D7**			**D14**			**D28**		
	**N**	**Mean**	**SD**	**N**	**Mean**	**SD**	**N**	**Mean**	**SD**	**N**	**Mean**	**SD**
WCC(cells/μL)	228	6414	2860	120	6767	2334	12	7321	3518	90	6678	2481
Ht (%)	376	38.3	5.54	271	35.1	5.58	11	36.2	3.82	218	37.4	5.12
ALT (U/L)	257	24.1	16.9	194	17.9	25.9	16	9.3	4.74	162	13.8	11.6
AST (U/L)	285	44.5	40.4	194	23.5	35.4	46	22.8	10.7	163	22.6	19.5
bilirubin (mg/dL)	155	0.61	0.60	110	0.30	0.23	45	0.43	0.20	66	0.46	0.49
creatinine (mg/dL)	237	0.67	0.34	168	0.81	1.19	46	053	0.12	165	0.71	0.29

Legend: WC = White Blood cell total Counts; Ht = haematocrit; ALT = alanine transaminase; AST = aspartate transaminase.

**Table 9:** Shift table in CTC (Common Toxicity Criteria) grades between Day 0–7 and Day 0-28

		**D7**					**D28**				
**DO CTC Grade**		**Grade 0**	**Grade 1**	**Grade 2**	**Grade 3**	**Total**	**Grade 0**	**Grade 1**	**Grade 2**	**Grade 3**	**Total**
WCC CTC Grade	Grade 0	92	5	2	3	102	70	7	0	1	78
	Grade 1	5	2	1	0	8	4	1	0	0	5
	Grade 2	4	1	1	0	6	2	0	1	1	4
	Grade 3	0	0	1	0	1	0	0	0	0	0
	total	101	8	5	3	117	76	8	1	2	87
AST CTC Grade	Grade 0	103	31	11	1	146	76	41	12	0	129
	Grade 1	11	14	6	1	32	3	6	9	1	19
	Grade 2	0	0	2	0	2	1	0	0	0	1
	Grade 3	0	0	1	0	1	0	0	0	0	0
	total	114	45	20	2	181	80	47	21	1	149
ALT CTC Grade	Grade 0	141	29	1	0	171	113	29	1	0	143
	Grade 1	4	5	1	0	10	1	3	0	0	4
	Grade 2	0	0	0	0	0	0	1	0	0	1
	Grade 3	0	1	0	0	1	0	0	0	0	0
	total	145	35	2	0	182	114	33	1	0	148
Creatinine CTC Grade	Grade 0	53	13	0	0	66	36	14	0	0	50
	Grade 1	7	4	0	0	11	7	1	0	0	8
	Grade 2	0	0	0	0		0	0	0	0	0
	Grade 3	0	0	0	0		0	0		0	0
	total	60	17	0	0	77	43	15	0	0	58
Bilirubin CTC Grade	Grade 0	82	7	1	1	91	42	5	3	1	51
	Grade 1	0	0	0	0	0	1	0	0	0	1
	Grade 2	0	0	0	0	0	2	0	1	0	3
	total	82	7	1	1	91	45	5	3	1	54

## Discussion

There is little safety information on ACT outside clinical trials, when used in real-life settings. The availability of such information, though, is important for policy decisions; while it may be difficult to tell the different forms of ACT apart based on efficacy, better knowledge of their safety profiles, along with other practical considerations, may help guide decisions.

Pharmaco-vigilance (PV) systems are variably established in malaria-endemic countries where ACT is the first-line malaria treatment, and so far, have failed to produce information in significant amounts [10]. PV is intended to generate signals about rare events but is less informative on the more common toxicities; typically, the absence of denominators makes it difficult, if not impossible, to estimate risks with some level of precision. Cohort event monitoring (CEM) can produce more complete information on events profiles, frequencies and associated risks factors, and this in real-life conditions. CEM is recommended by the WHO and has already been applied to ACT [5,6].

The present study generated information not only on the safety and tolerability of ASAQ, but also on practical aspects of setting up informative, quality and sustainable information systems in peripheral settings. Strengths and weaknesses are discussed below.

### Safety outcomes

The system set in place here provided for a set of pre-defined signs/symptoms to be assessed and graded before as well as after treatment. This proved very useful in differentiating disease- and treatment-related signs/symptoms. It revealed that 21% of the patients and 40% of the events that would have been reported as AEs were already present on admission.

Assessing intensity of events is difficult to teach and standardize across assessors, and could be a source of bias; this was however minimized by having the same person assessing a patient before and after treatment.

Overall, just under 10% of subjects had a total of almost 700 TESS (two per treated patient on average), three-quarters of which were mild or moderate. Children under six years of age were at lower risk of events (either pre-treatment of following treatment), but it is not clear if this finding merely reflects the challenge of obtaining reliable information from young children.

No toxicities emerged from the clinical laboratory evaluation. The incidence of SAEs (including death) was five per thousand; though, for comparison, about one-third of the rate revealed from a meta-analysis of randomized controlled trials of ASAQ in research settings [11], this shows that the system was accurate enough to capture serious events.

Concerning rare events, 49 cases of extra-pyramidal reaction following administration of ASAQ are reported to the Upssala Monitoring centre, including two cases of tongue protrusion in adults and one in children. In this CEM, two cases of tongue protrusion were detected within a timeframe compatible with the other reported cases; both receded within 15 minutes after administering dexamethasone [12]. Such extrapyramidal events are generally imputed to AQ, but the mechanism is not known. Here, there was no enough information on possible concomitant intake of other medicines or traditional remedies to understand whether an interaction might have been involved.

With its ~3,700 records, one would be 80% confident that if this study detected an event, this will occur on average in at least five per 10,000. More rare, serious adverse events would require much larger numbers.

### Adequacy of dosing

Records collected allowed assessing adequacy of dosing as administered by nurses or health workers at peripheral health centres following treatment recommendations and manufacturers’ instructions. Treatment was administered based on the patient weight for the loose combination, or age for the co-blistered and fixed-dose products; of note, both the tablet dosage and the age groups differed between these two forms – which explains the differences observed. Age-based dosing with the fixed-dose product was as accurate (97% and 82% of treatments were within the therapeutic ranges for AS and AQ, respectively) as weight-based dosing with the loose, individually-formulated products (93% and 86%). The total dose of AQ was also ~20% higher with the fixed than the loose combination.

The co-blistered product was clearly less amenable to dosing patients accurately than the other two, and there was indirect evidence that dosing errors (42% and 48% overdosing for AS and AQ, respectively) would be less tolerated (see below) as also implied in a previous study in the same setting [8]. The fix-dose product is now the recommended formulation, and at the time of writing the only form available for treating malaria in Senegal.

### Risks

One of the limitations of this study is that it did not allow estimating specific risks related e.g. to age or products. The reasons are that, when analysing the results, it became obvious that multiple interactions existed between variables such as the year of study, the product and the patient age. One should bear in mind that this study was conducted over a nine-year period that witnessed fundamental changes in malaria in this district: the product form used changed from loose, co-blistered, fix combination products, with overlaps between products, prevalence dropped and patient’s age increased significantly [13]. Furthermore, reporting was not uniformly applied by health workers and nurses and over time (see below). More information was gathered on exposure to the loose (49% of ASAQ treatments enrolled in this study) and co-blistered (42%) products, and much less on the fixed product (9%), and effects were confounded by evolving age and variable health workers’ adherence to the reporting system.

It was possible, however, to quantify the risk of experiencing a toxic event (TESS) in relation to dosage by year of study. Over-dosage doubled the risk of TESS over the entire study period, but this was statistically significant only during 2003–2007, which are the years when the co-blister was either the predominant or the only product in use.

### Feasibility aspects of safety monitoring

This CEM study was purposely nested within the daily routine work of the health centres. Apart from the eligibility criteria, there was no special procedure or randomization list for selecting patients to recruit into the CEM study. The nurses were to enroll as many patients as they could possibly afford within their capacity and considering their various daily commitments. The reason behind this was that it would provide useful information on the feasibility of CEM at peripheral health centres as part of daily routine. Indeed, this could have introduced a selection bias, but again, this will be an inherent feature of any CEM.

Approximately one in four (26%) of all the patients receiving ASAQ during 2001–2009 was recruited into this CEM programme, which is a reasonable proportion. However, the programme was not uniformly applied; the proportion fluctuated with time (4-99% per annum) and the facility (2-61%); 50% of the cases had been enrolled by the end of 2003. There are reasons for that. Nurses and community health workers (all literate) were trained (and retrained) to collect and record the data. At the beginning, adherence was very high. However, this was an additional task on top of their routine work, and with increasing workload while scaling-up the implementation of RDT plus ACT, the proportion of patients enrolled in the safety monitoring fell – to raise again when numbers started decreasing again. There was a clear inverse correlation between the number of patients treated with ASAQ, the proportion of these enrolled in the safety survey, and the proportion of patients reported to have events. This is exemplified by the surprisingly low (one-third) and variable accuracy of reporting on presentation, which ranged from less than 2% to almost 98% of patients (all with parasitologically-confirmed malaria) having signs/symptoms pre-treatment – which was inversely proportional to the number of patients enrolled. Workload was a recognized deterrent also for PV in other settings [14]. This means that under-reporting and inconsistent reporting of events could not be ruled out; however, it is reassuring to see that from 2007, > 90% of patients are reported to have events pre-treatment. This may be explained by the fact that recently not only the number of cases seen (workload) has decreased, but also the age of patients has increased (now patients are older, thus easier to interrogate and obtain information). Human (motivation was not the same for all) and structural factors (staff turn-over) are important determinants, too.

There are also costs involved, related to training (and re-training), quality control, reporting and analysis. Incentives may be considered to motivate staff to carry out these additional activities on top of their daily work. Costs will increase especially if laboratory tests are added. The problem is that clinical signs and symptoms will not always reveal all toxicities, some of which may be clinically silent. Examples are asymptomatic neutropaenia and hepatitis which have been described for AQ [15] and AS [16]. For a system to be more fully informative, laboratory tests should be included at least on a proportion of patients and targeted to detect known toxicities, but the costs may be prohibitive for resource-constrained settings.

In addition, there are political willingness, and (international and country) regulations. Post-marketing surveillance and pharmaco-vigilance are mandatory for manufacturers, but this does not work well in unregulated markets (where products are generics available through the private and the informal sectors), and in countries with no or suboptimal systems, as is the case in many malaria-endemic countries. No one system alone will provide reliably all the information needed. CEM is recommended by the WHO and countries should consider adopting it. However, it has advantages and disadvantages. As a pre-requisite, feasibility studies like this one should be set in place, along with realistic costing estimates of setting it up and sustaining it.

Data quality is paramount for the information to be reliable; quality checks must be run on the performance of the system. Unless minimum requirements are met, investing in such a system would not be justified. In this study, due to budget limitations, it was not possible to provide for quality control, a limitation which is clearly reflected in the abovementioned erratic performance, and which prevented more in-depth analyses. At the same time, the absence of such a system allowed to bring out practical issues that will require corrective actions, should a similar system be set in place.

Lastly, it should be clear, both at country and international level, that collecting and storing information is not enough, if not followed up by developing an updated risk-management plan informed by large databases collating data from different locations for individual drugs and ACT in general.

### Overall study evaluation

(i) The study was successful in characterizing known reactions. In particular, it was possible to quantitate frequencies (both numerator and denominator available). The size of the cohort was adequate for known reactions but probably not enough to generate a signal on yet unrecognized, rare toxicities. While lacking a comparator drug, this study provides valuable benchmarking for future assessments both locally and elsewhere, as well as for risk management.

(ii) Collecting data on the occurrence and intensity of a set of signs/symptoms pre-treatment as well as post-treatment made it possible to reduce the background noise (generated by malaria itself and individual factors) and improve the quality and specificity of the signal (possible treatment-induced events).

(iii) Data were collected that allowed assessing the adequacy of dosing with different ASAQ formulations, and analyse its effects on tolerability.

(iv) The study also generated information that could be used to improve performance. For instance, staff’s compliance was on average satisfactory (one-fourth of all ASAQ treatments enrolled) but highly variable (also in terms of completeness of information)– which may have introduced a number of biases and confounders. Higher consistency in needed. In that respect training and quality control systems will be required.

(v) The questions that remain to be answered is whether a system like this is sustainable and under which conditions, and whether it is applicable elsewhere. Research should be done into the conditions that would make it cost-effective, including the amount and quality of data generated, and the use thereof for decision-making possibly in parallel with classical passive pharmaco-vigilance activities.

## Competing interest

No competing interest to declare.

The opinions expressed in this paper are those of the authors and may not reflect those of their employing organizations. P. Olliaro is a staff member of the WHO; M. Vaillant is a staff member of CRP-Santé; the authors alone are responsible for the views expressed in this publication and they do not necessarily represent the decisions, policy or views of the WHO or CRP-Santé.

## Authors’ contributions

PB contributed to conception and design, acquisition of data, analysis and interpretation of data; drafted the manuscript, revised it critically for important intellectual content. MV contributed to conception and design, acquisition of data, analysis and interpretation of data; drafted the manuscript, revised it critically for important intellectual content. PO contributed to conception and design, acquisition of data, analysis and interpretation of data; drafted the manuscript, revised it critically for important intellectual content. All authors gave final approval of the version to be published. Each author takes public responsibility for appropriate portions of the content.
